# Real space iterative reconstruction for vector tomography (RESIRE-V)

**DOI:** 10.1038/s41598-024-59140-1

**Published:** 2024-04-25

**Authors:** Minh Pham, Xingyuan Lu, Arjun Rana, Stanley Osher, Jianwei Miao

**Affiliations:** 1 Department of Physics and Astronomy and California NanoSystems Institute, University of California, Los Angeles, CA 90095 USA; 2 Department of Mathematics, University of California, Los Angeles, CA 90095 USA; 3 Institute of Pure and Applied Mathematics, University of California, Los Angeles, CA 90095 USA; 4 School of Physical Science and Technology, Soochow University, Suzhou, 215006 China

**Keywords:** Optics and photonics, Optical physics, Applied mathematics

## Abstract

Tomography has had an important impact on the physical, biological, and medical sciences. To date, most tomographic applications have been focused on 3D scalar reconstructions. However, in some crucial applications, vector tomography is required to reconstruct 3D vector fields such as the electric and magnetic fields. Over the years, several vector tomography methods have been developed. Here, we present the mathematical foundation and algorithmic implementation of REal Space Iterative REconstruction for Vector tomography, termed RESIRE-V. RESIRE-V uses multiple tilt series of projections and iterates between the projections and a 3D reconstruction. Each iteration consists of a forward step using the Radon transform and a backward step using its transpose, then updates the object via gradient descent. Incorporating with a 3D support constraint, the algorithm iteratively minimizes an error metric, defined as the difference between the measured and calculated projections. The algorithm can also be used to refine the tilt angles and further improve the 3D reconstruction. To validate RESIRE-V, we first apply it to a simulated data set of the 3D magnetization vector field, consisting of two orthogonal tilt series, each with a missing wedge. Our quantitative analysis shows that the three components of the reconstructed magnetization vector field agree well with the ground-truth counterparts. We then use RESIRE-V to reconstruct the 3D magnetization vector field of a ferromagnetic meta-lattice consisting of three tilt series. Our 3D vector reconstruction reveals the existence of topological magnetic defects with positive and negative charges. We expect that RESIRE-V can be incorporated into different imaging modalities as a general vector tomography method. To make the algorithm accessible to a broad user community, we have made our RESIRE-V MATLAB source codes and the data freely available at https://github.com/minhpham0309/RESIRE-V.

## Introduction

Tomography has had a radical impact on diverse fields ranging from medical diagnosis^1^ to 3D structure determination of proteins^2^, crystal defects^3,4^, amorphous materials^5,6^ at the atomic resolution. Despite its diverse applications, the central problem in tomography remains the same, that is, how to accurately reconstruct the 3D structure of an object from many projections with noise and incomplete data. Tomography reconstruction algorithms can be categorized into three types: (1) a direct inversion method - filtered back projection (FBP)^1,2^, (2) real space or Fourier-based iterative methods, and (3) deep learning related algorithms^7^. Here we primarily focus on the second type. Conventional iterative algorithms include algebraic reconstruction technique (ART)^8^, simultaneous algebraic reconstruction technique (SART)^9^, simultaneous iterative reconstruction technique (SIRT)^10,11^, and nonuniform fast Fourier transform (NUFFT)^12^. These algorithms can incorporate mathematical regularizations such as total variation (TV)^13^ and Model-based iterative reconstruction (MBIR)^14^. In recent years, more advanced iterative algorithms, which are inspired by iterative phase retrieval in coherent diffractive imaging^15,16^, have been developed, including equal slope tomography (EST)^17,18^, generalized Fourier iterative reconstruction (GENFIRE)^19,20^, and real space iterative reconstruction (RESIRE)^5,21^.

In particular, RESIRE, using the Radon transform as the forward projection and the Radon transpose as the back projection, is superior to other existing tomographic algorithms^21^. Furthermore, RESIRE can not only work with multiple tilt axes, extended objects, partially blocked projections, and large missing wedges, but also improve the tilt angle precision by implementing angular refinement. RESIRE has been used to determine the 3D atomic structure of amorphous materials^5,6^, heterogeneous nanocatalysts^22^, the chemical order and disorder in medium/high entropy alloys^23^ and 3D nanoscale imaging of mesoporous structure^24^. Despite these applications, they only deal with scalar tomography, where each voxel in a 3D reconstruction has a magnitude but no direction. However, in some important applications vector tomography is required, where each voxel has a magnitude and a direction such as the electric and magnetic field.

Over the years, several vector tomography reconstruction methods have been developed, including vector electron tomography with Lorentz transmission electron microscopy and holography^25–32^, soft and hard x-ray vector tomography^15,33–41^. In particular, the combination of ptychography, a powerful coherent diffractive imaging method^16,42^, and vector tomography can in principle achieve the highest spatial resolution, which is only limited by the wavelength and the diffraction signal^34,38,41^. Very recently, we have merged soft-x-ray magnetic circular dichroism and ptychography with vector tomography to image the 3D topological magnetic monopoles and their interaction in a ferromagnetic meta-lattice with a spatial resolution of 10 nm^41^. We have also applied it to observe the topological magnetic order in superparamagnetic nanoparticles self-assembled at the liquid-liquid interface^43^.

Here, we present the mathematical foundation and implementation of the real space iterative reconstruction algorithm for vector tomography (RESIRE-V), which represents an important advance over RESIRE for scalar tomography. RESIRE-V can accurately reconstruct the 3D magnetization vector field from multiple tilt series each with a limited number of experimental projections^41,43^. Furthermore, due to the experimental error, the measured tilt angles may not always coincide with the true orientations of the projections. To tackle this problem, we further implement an iterative angular refinement procedure to reduce the tilt angle error^21^, enabling us to obtain more accurate vector tomographic reconstruction. Both numerical simulations and experimental data have been used to demonstrate the effectiveness of our method. In addition, we provide an analysis of the vector tomography reconstruction, requirement, and robustness.

## Methods

**Figure 1:**
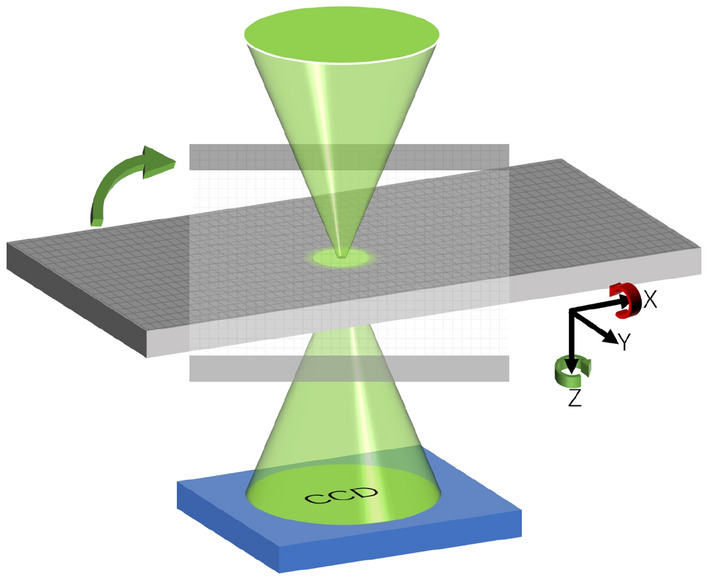
Schematic of sample rotation geometry in vector tomography. An X-ray beamline with left- or right-circular polarization is focused onto a sample, which can be rotated around the X axis (green arrow), termed the in-place rotation. At each in-plane rotation angle, a tilt series is acquired by rotating the sample around the X axis (red arrow). At each tilt angle, the focused beam scans across the sample and two sets of diffraction patterns with left- and right-circular polarization are collected by a charge-coupled device (CCD) detector.

We begin with some setup and conventions. First, we employ Euler angles to describe the orientation of a rigid body with respect to a fixed coordinate system. For example, the orientation representation ZYX used intensively in our research fits well with vector tomography experiments: samples are rotated about the Z-axis (in-plane rotation) before a set of tilt series (rotation about the Y-axis) are acquired, where the in-plane-rotation is defined as the rotation of the samples in the XY plane, i.e. around the Z-axis of the beam direction (Fig. 1). The last rotation about the X-axis is helpful in angular refinement. We use the notation $Z_{\phi }Y_{\theta }X_{\psi }$ to represent Euler angle rotations: the first rotation is about the Z-axis by an angle $\phi$, followed by a rotation about the Y-axis by an angle $\theta$, and ends with a rotation about the X-axis by an angle $\psi$, respectively. The corresponding rotation matrix $R_{Z_{\phi }Y_{\theta }X_{\psi }} = R_{\phi }^Z \, R_{\theta }^Y \, R_{\psi }^X$ is defined to be the product of three single-axis rotation matrices about the Z, Y, and X axes by angles $\psi$, $\theta$ and $\phi$ respectively:

$$\begin{aligned} R_{\phi }^Z := \begin{bmatrix} \cos \phi &{} -\sin \phi &{} 0\\ \sin \phi &{} \cos \phi &{} 0 \\ 0 &{} 0 &{} 1 \end{bmatrix} ,\quad R_{\theta }^Y := \begin{bmatrix} \cos \theta &{} 0 &{} \sin \theta \\ 0 &{} 1 &{} 0 \\ -\sin \theta &{} 0 &{}\cos \theta \end{bmatrix} , \quad R_{\psi }^X := \begin{bmatrix} 1 &{} 0 &{} 0 \\ 0 &{} \cos \psi &{} -\sin \psi \\ 0 &{} \sin \psi &{} \cos \psi \end{bmatrix} \end{aligned}$$

For short notation, we write $R_{\Theta }$ instead of $R_{Z_{\phi }Y_{\theta }X_{\psi }}$ where $\Theta = \{ \phi , \theta , \psi \}$ (no orientation is specified). In perfect experimental conditions where there is no X-axis rotation, $\psi$ is zero. Otherwise, $\psi$ can be non-zero and we use angular refinement to determine $\psi$. The convention finishes and we move to the formulation part.

### Formulation

For an x-ray beam propagating along the z direction (standard unit vector $\vec {e}_z = [0, \, 0, \, 1]^T$), only the z component of the magnetization contributes to the 2D signals. The contribution takes either positive or negative values depending on the left or right circular polarization. In the case of rotation, we need the inner product $\big \langle R_{\Theta } \, {\textbf {M}}(R_{\Theta }^{\dagger } \vec {r}) , \, \vec {k}\big \rangle$ to count for the contribution. Here, ${\textbf {M}}= [M_x, \, M_y, \, M_z ]$ is the magnetization vector field, which is a function of the Cartesian coordinate vector $\vec {r}= ( x, \, y, \, z )$, and $R_{\Theta }^\dagger$ is the adjoined, and also inverse and transpose, of $R_{\Theta }$. Adding the non-magnetic term *O* and taking the integral along the z axis (projection), we obtain the 2D signal:

1 $$\begin{aligned} \int _z c \big \langle R_{\Theta } \, {\textbf {M}}(R_{\Theta }^\dagger \vec {r}) , \vec {e}_z \big \rangle + O (R_{\Theta }^{\dagger } \vec {r})\, dz = P_{\Theta } \end{aligned}$$

where *c* is a constant that relates the XMCD signal to the magnetization and the pixel size. We can temporarily let ${\textbf {M}}$ absorb *c* in the derivation for simplicity and then rescale ${\textbf {M}}$ after the reconstruction. We then write this equation using the change of variable $\vec {r}\leftarrow R_{\Theta }^{\dagger } \vec {r}$:

2 $$\begin{aligned} \int _{L_{\Theta }} \big \langle {\textbf {M}}(x,y,z) , R_{\Theta }^{\dagger } \vec {e}_z \big \rangle + O ( x,y,z )\, dz = P_{\Theta } (x,y) \end{aligned}$$

Rotating the sample by some Euler angles $\Theta$ and taking the integral along the z-axis is equivalent to taking the line integral along the opposite rotation direction (passive rotation). To solve this equation numerically, we need to discretize the equation. Replacing the line integral with a projection operator and expanding the inner product, we represent the equation algebraically:

3 $$\begin{aligned} \Pi _{\Theta } \Big ( \alpha _{\Theta } M_x + \beta _{\Theta } M_y + \gamma _{\Theta } \, M_z + O \Big ) = P_{\Theta } \end{aligned}$$

where $\Pi _{\Theta }$ is the projection operator and $P_{\Theta }$ is the corresponding projection with respect to Euler angles $\Theta = (\phi , \, \theta , \, \psi )$. In this notation, we drop the spatial variables (*x*, *y*, *z*) for simplicity. Let $\vec {n}_{\Theta } = [\alpha _{\Theta }, \, \beta _{\Theta }, \, \gamma _{\Theta }]$ be the last column of $R_{\Theta }^\dagger$. Specifically, if we use the orientation representation $Z_{\phi }Y_{\theta }$, then the normal vector is given by: $\vec {n}_{\Theta } = [\alpha _{\Theta }, \, \beta _{\Theta }, \, \gamma _{\Theta }] = [\sin \theta \, \cos \phi , \, \sin \theta \, \sin \phi , \, \cos \theta ]$.

One can verify that the second magnetization component does not contribute to the measured projections when $\phi =0$. It implies that other types of rotation are required for successful vector tomography reconstructions. Since $\Pi _{\Theta }$ is linear, we can apply the commutative property and distribute the linear operator to each magnetization component:

4 $$\begin{aligned} \alpha _{\Theta } \, \Pi _{\Theta } (M_x) + \beta _{\Theta } \, \Pi _{\Theta } (M_y) + \gamma _{\Theta } \, \Pi _{\Theta } (M_z) +\Pi _{\Theta } (O) = P_{\Theta } \end{aligned}$$

Equation 4 describes that the three 3D magnetization components and the non-magnetic structure are coupled via a linear constraint. So far, we have formulated the vector tomography in perfect condition (noise free). In the presence of noise and assuming that the noise is Gaussian with mean 0 and variance $\sigma ^2$, we add the noise term ${\mathcal {N}}(0,\sigma ^2)$ to the left-hand side of the equation.

5 $$\begin{aligned} \alpha _{\Theta } \, \Pi _{\Theta } (M_x) + \beta _{\Theta } \, \Pi _{\Theta } (M_y) + \gamma _{\Theta } \, \Pi _{\Theta } (M_z) +\Pi _{\Theta } (O) + {\mathcal {N}}(0,\sigma ^2) = P_{\Theta } \end{aligned}$$

We first denote $P_{\Theta }^+$ and $P_{\Theta }^-$ are random variables, with the same variance $\sigma ^2$, that represent the left and right polarized projections by Euler angles $\Theta$ respectively. Let $b_{\Theta }^- = \frac{1}{2} \big ( P_{\Theta }^+ - P_{\Theta }^- \big )$ be a random variable as describe, we obtain a simpler linear equation:

6 $$\begin{aligned} \alpha _{\Theta } \, \Pi _{\Theta } (M_x) + \beta _{\Theta } \, \Pi _{\Theta } (M_y) + \gamma _{\Theta } \, \Pi _{\Theta } (M_z) + {\mathcal {N}}\left( 0,\frac{\sigma ^2}{2}\right) = b_{\Theta }^- \end{aligned}$$

Note that, by the law of large numbers, taking the average of two random variables with the same mean and variance results in a new random variable where the mean stays the same, but the variance gets reduced by half^44^. We can use maximum likelihood estimation to recover three-dimensional magnetization from corrupted 2D signals. Specifically for Gaussian noise, the log maximum likelihood function is the sum of the squared errors (or $l_2$ distances) between the desired and measured signals:

7 $$\begin{aligned} \min _{{\textbf {M}}} \, \varepsilon ({\textbf {M}})&= \frac{1}{2}\sum _{\Theta } { \big \Vert \alpha _{\Theta } \, \Pi _{\Theta } (M_x) + \beta _{\Theta } \, \Pi _{\Theta } (M_y) + \gamma _{\Theta } \, \Pi _{\Theta } (M_z) - b_{\Theta }^- \big \Vert ^2 } \end{aligned}$$

We can always write the minimization problem in the form $\varepsilon ({\textbf {M}}) = \frac{1}{2}\sum _{\Theta } { \big \Vert \Pi _{\Theta } \big ( \alpha _{\Theta } M_x + \beta _{\Theta } \, M_y + \gamma _{\Theta } \, M_z \big ) - b_{\Theta }^- \big \Vert ^2 }$ thanks to the linearity of the projection operator. For efficient implementation, the latter form is preferred over Eq. 7.

The maximum likelihood function will appear different for other types of noise; however, the famous least-squares form can still handle other circumstances because of its simplicity and effectiveness. Equation 7 is our final form of vector tomography formulation. To recap, we highlight our innovation:

- Before this work, Eq. 4 is used to solve the 3D magnetization vector field^34,35,38^.
- In our new approach, we use left and right polarization to derive Eq. 6. RESIRE is then used to reconstruct the scalar object from which the support is evaluated.
- We solve the minimization 7 for the three magnetic components using the above support as a constraint.

Separating the reconstruction into the above two steps makes the result more accurate. The remaining part is designing a numerical scheme to solve the minimization 7.

### RESIRE-V algorithm

We develop our algorithm based on the real space iterative technique. Noticing that the projection operator is linear, one can construct a matrix representation for each $\Pi _{\Theta }$. We assume that the projections have size $n\times n$, and the sample gets reconstructed with thickness *n*. In that case, each projection matrix $\Pi _{\Theta }$ has $O(n^3)$ non-zero elements. In the case of over-constraint, Eq. 7 can be solved using the normal equation. Otherwise, in the case of under-determined system, we need to add a regularizer to prevent overfitting. When adding a damping term as a regularizer, we have an overconstrained system again and we can solve the equation using the normal equation as in the over-constraint case. In either case, storing projection matrices is tremendously expensive since the size expands in the cubic order of the projection size. Here, to save memory usage, we do not need to store the projection matrices but compute the forward projections at every iteration instead. This procedure will increase the number of computations; however, GPU parallel computing can help reduce the computational time significantly.

Our gradient descent algorithm incorporates two steps: forward projection and back projection. For the first step, we institute our 3D Radon transform using the idea of 2D Radon transform which can be found elsewhere^45,46^. The algorithm first divides pixels in a 3D image into four sub-pixels and projects each sub-pixel individually. Specifically, at a tilt angle, we compute the corresponding coordinate of each pixel and project it on the XY plane. The value of each sub-pixel is distributed proportionally to the four nearest neighbors, according to the distance between the projected location and the pixel centers.

For example, consider that the pixel projection hits a position (*x*, *y*), and let $\lfloor x \rfloor$ and $\lfloor y \rfloor$ be the largest integers less than *x* and *y*, respectively. Then the four nearest bins, centering at $(\lfloor x \rfloor , \lfloor y \rfloor )$, $(\lfloor x \rfloor , \lfloor y \rfloor + 1)$, $(\lfloor x \rfloor + 1, \lfloor y \rfloor )$ and $(\lfloor x \rfloor +1, \lfloor y \rfloor + 1)$, take $(1 + \lfloor x \rfloor - x) (1 + \lfloor y \rfloor - y)$, $(1 + \lfloor x \rfloor - x) (y - \lfloor y \rfloor )$, $(x - \lfloor x \rfloor ) (1 + \lfloor y \rfloor - y)$ and $(x - \lfloor x \rfloor ) (y - \lfloor y \rfloor - x)$ values of the pixel, respectively. Hence, if the pixel projection hits the center point of a bin, the bin on the axis gets the entire value of the pixel. In the specific case where the pixel projection hits the border between four bins, the pixel value is split evenly between these four bins. Other techniques to compute the contribution of the value of a pixel projection can be found elsewhere^47^. Illustration of 2D Radon transform can be found in the Supplementary Fig. 1.

Next, we establish the transpose of the Radon transform for the back-projection step. This process is similar to the forward projection but in reverse order. According to the distance between the projected location and the pixel centers, the four nearest neighbors to a projection sub-pixel proportionally contribute their values to the sub-pixel. If the pixel projection hits the border between 4 bins, the pixel takes a quarter value of each of these four bins. Our sub-pixel division and linear interpolation are efficient with the complexity $O( N^3)$ and can be highly parallelized using GPU cuda programming. Other forward and back-projection techniques with higher accuracy, such as separable footprint^47^ and gram filtering^48,49^, can be considered. With these methods, there will be a trade-off between accuracy and computational cost/memory usage. Error analysis on the discretization and projection operator can also be found elsewhere^48,49^.

After specifying the forward and back projection, we can now take the gradient of the error metric $\varepsilon ({\textbf {M}})$ in Eq. 7 with respect to each magnetization component:

8 $$\begin{aligned} \frac{\partial \varepsilon }{\partial M_x}&= \sum _{\Theta } \alpha _{\Theta } \, \Pi _{\Theta }^T \, \Big ( \alpha _{\Theta } \, \Pi _{\Theta } (M_x) + \beta _{\Theta } \, \Pi _{\Theta } (M_y) + \gamma _{\Theta } \, \Pi _{\Theta } (M_z) - b_{\Theta }^- \Big ) \nonumber \\&= \sum _{\Theta } \alpha _{\Theta } \, \Pi _{\Theta }^T \Big ( \Pi _{\Theta } \big ( \alpha _{\Theta } M_x + \beta _{\Theta } \, M_y + \gamma _{\Theta } \, M_z \big ) - b_{\Theta }^- \Big ) \end{aligned}$$

where $\Pi _{\Theta }^T$ is the transpose operator of the Radon transform for Euler angles $\Theta$. As mentioned above, the second form of the gradient will be used for the C++/Cuda implementation.

Next, we show that the gradient is L-lipschitz and the algorithm will converge to the global minimum with an appropriate step size. Specifically, we want to find an L such that the following inequality is true:

9 $$\begin{aligned} \big \Vert \nabla \varepsilon ({\textbf {M}}_1) - \nabla \varepsilon ({\textbf {M}}_2) \big \Vert \le L \big \Vert {\textbf {M}}_1 - {\textbf {M}}_2 \big \Vert \quad \forall \, {\textbf {M}}_1, \, {\textbf {M}}_2 \end{aligned}$$

**Figure 2:**
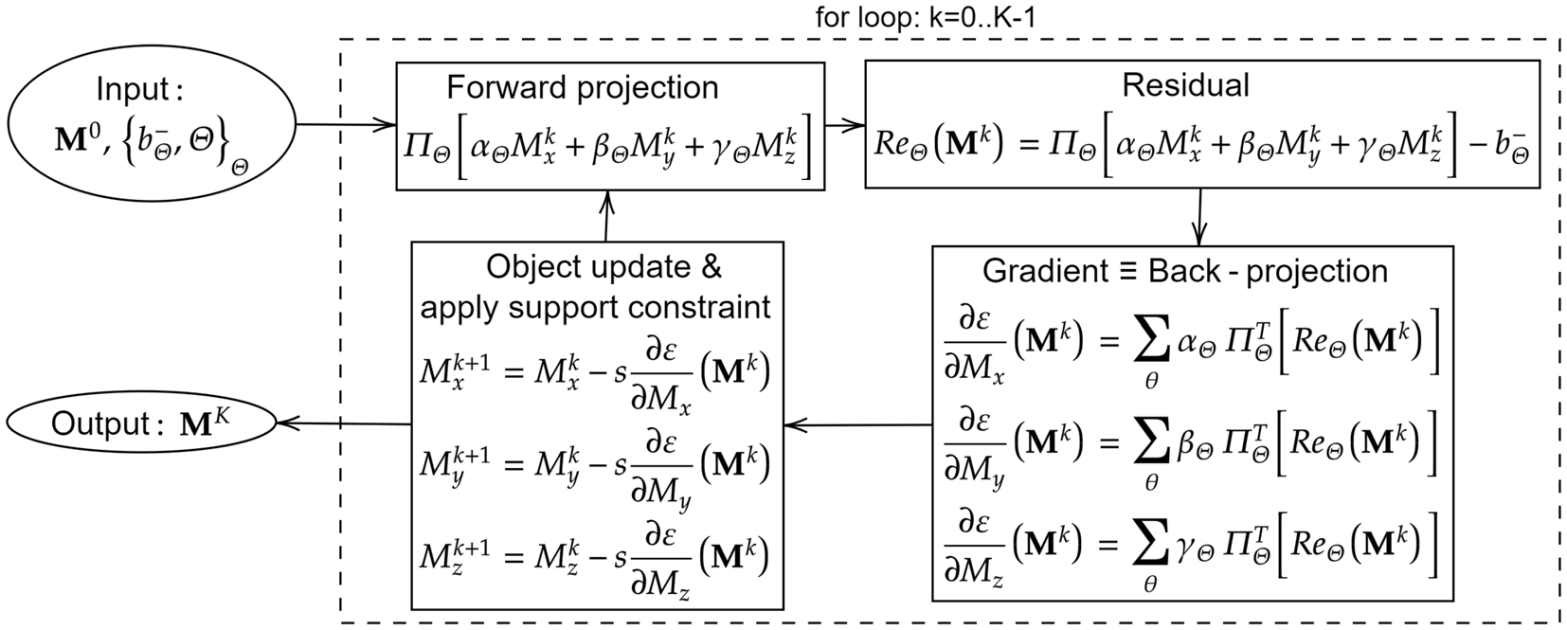
RESIRE-V diagram: Inputs are the differences between the left and right polarization projection and the support from the scalar reconstruction. The algorithm uses a for loop to refine the magnetization vector field ${\textbf {M}}$. At each iteration, it calculates the forward projections and computes their differences with the measured ones. The residuals (or differences) are back-projected to yield gradients. The algorithm will use these gradients to update the magnetization and apply the support constraint. The step size $\frac{t}{\sqrt{3}nN}$ is replaced by *s* for simplification purposes.

The Lipchitz constant L gets calculated as $\sqrt{3} n N_z$ where *n* and $N_z$ are the number of projections and the thickness in pixels of the reconstruction, respectively. Hence, we can choose the step size to be 1/*L* for the convergence guarantee. Details of the proof can be found in the Supplementary, step-size analysis. The algorithm is finalized and described step by step in pseudocode 1 and Fig. 2.

**Algorithm 1:**
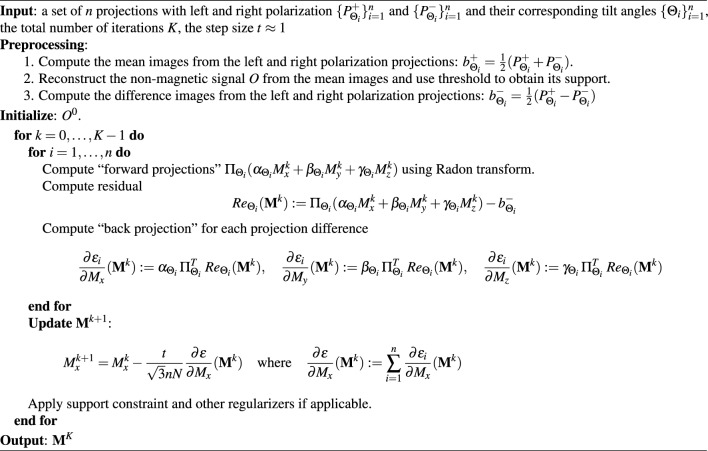
RESIRE-V

For efficient implementation, the gradient w.r.t. each component $\frac{ \partial \varepsilon }{\partial M_x} = \sum _{i} \frac{\partial \varepsilon _i}{\partial M_x}$ will be accumulated. In addition, the step size is generalized to be $\frac{t}{ \sqrt{3} n N}$ where $t \approx 1$ is the normalized step size. According to our analysis, *t* should be less than or equal to 1 for the convergence guarantee. The analysis uses triangle inequalities and considers the worst-case scenario. In practice where better scenarios are more popular, the algorithm can converge with *t*’s values slightly larger than 1. The analysis is complete, and we move to the discussion on conditions for vector tomography reconstruction.

### Analysis: conditions for vector tomography reconstruction

Scalar tomography is a well-posed problem and can obtain a faithful reconstruction from single-axis tilt series, provided the Nyquist-Shannon sampling theorem is satisfied. But 3D vector tomography has three scalar components to be reconstructed, thus requiring more stringent data acquisition schemes.

In the 1980s, Norton showed that the reconstruction of a diverge-less 2D vector field appeared to be unique^50^. Prince gave a more generalized discussion of the reconstructions of arbitrary vector fields in the 1990s. He demonstrated that, for reconstructing an arbitrary *n*-dimensional vector field, *n* tomographic projection datasets in which the probe is sensitive to n different directions of the vector field needed to be acquired^51,52^. The idea of using more than one tilt rotation axes has been used successfully in scalar tomography to reduce the missing wedge artifacts^53,54^. That idea is also believed to be a key to solving the vector tomography problem.

In our research, we use the Fourier slice theorem to show specific experimental conditions for the reconstruction of arbitrary vector fields. The theorem states that the 2D Fourier transform of a 2D projection equals a 2D slice through the origin of the 3D Fourier transform of an object. The 2D slice is defined based on the corresponding rotation angle. In the case of noise-free, we apply the Fourier transform to both sides of Eq. 6.

10 $$\begin{aligned}{} & {} \alpha _{\Theta } \, {\mathcal {F}} \big [ \Pi _{\Theta } (M_x) \big ] + \beta _{\Theta } \, {\mathcal {F}} \big [ \Pi _{\Theta } (M_y) \big ] + \gamma _{\Theta } \, {\mathcal {F}} \big [ \Pi _{\Theta } (M_z) \big ] = {\mathcal {F}} \big [ b_{\Theta }^- \big ] \end{aligned}$$

Applying the Fourier slice theorem, we have a linear constraint involving the Fourier transforms ${\hat{m}}_x$, ${\hat{m}}_y$ and ${\hat{m}}_z$ of the three magnetization components $M_x$, $M_y$ and $M_z$. This constraint applies to every Fourier point $\vec {\xi }$ on a 2D Fourier slice through the origin.

11 $$\begin{aligned}{} & {} \alpha _{\Theta } \, {\hat{m}}_x ( \vec {\xi }) + \beta _{\Theta } \, {\hat{m}}_y ( \vec {\xi }) + \gamma _{\Theta } \, {\hat{m}}_z ( \vec {\xi }) = {\hat{b}}_{\Theta }^- (\vec {\xi }) \quad \text {where} \quad \langle \vec {\xi }, \vec {n}_{\Theta } \rangle \nonumber \\{} & {} \quad = 0 \text {, and } \vec {n}_{\Theta } = [\alpha _{\Theta }, \, \beta _{\Theta }, \, \gamma _{\Theta } ] \end{aligned}$$

The extra constraint $\langle \vec {\xi }, \vec {n}_{\Theta } ) = 0$ is required by the requirement that a point belongs to a plan through the origin if the inner product between $\vec {\xi }$ and the normal vector of the plan is zero. If sufficient measurements are provided, one can sample the values of all Fourier points on the frequency domain and we can extend Eq. 11 to every point $\vec {\xi }$ on the 3D Fourier domain.

In order to separate ${\hat{m}}_x(\vec {\xi })$, ${\hat{m}}_y(\vec {\xi })$ and ${\hat{m}}_z(\vec {\xi })$ for a given 3D frequency point $\overrightarrow{\xi }$, we need to find three 2D Fourier slices whose normal vectors $\vec {n}_{\Theta }$ form a linear independent system in ${\mathbb {R}}^3$ and that go through the origin and contains $\overrightarrow{\xi }$. This is impossible since the set of normal vectors $\vec {n}_{\Theta }$ that satisfies the constraint $\langle \vec {\xi }, \vec {n}_{\Theta } \rangle = 0$ lies in a linear subspace of dimension two.

We give an example of in-plane rotations where the orientation is given by $Z_{\phi }Y_{\theta }$. Recalling that the normal vector corresponding to the in-plane rotation has the form $\vec {n}_{\Theta } = (\sin \theta \, \cos \phi , \, \sin \theta \, \sin \phi , \, \cos \theta )$, we can find infinitely many 2D slices that contain the point $\vec {\xi }=(1, \, 1,\, 1)$. For example, we name three projections with the corresponding Euler angles $(\phi _1, \, \theta _1) = (0^\circ , \,$-$45^\circ )$, $(\phi _2, \, \theta _2) = (120^\circ , \,$-$69.90^\circ )$ and $(\phi _3, \, \theta _3) = (-120^\circ , \, 36.21^\circ )$.

12 $$\begin{aligned} {\left\{ \begin{array}{ll} &{}\sin \theta _1 \, \cos \phi _1 \, {\hat{m}}_x(\vec {\xi }) + \sin \theta _1 \, \sin \phi _1 \, {\hat{m}}_y(\vec {\xi }) + \cos \theta _1 \, {\hat{m}}_z(\vec {\xi }) = {\hat{b}}_{\Theta _1}^-(\vec {\xi }) \\ &{}\sin \theta _2 \, \cos \phi _2 \, {\hat{m}}_x(\vec {\xi }) + \sin \theta _2 \, \sin \phi _2 \, {\hat{m}}_y(\vec {\xi }) + \cos \theta _2 \, {\hat{m}}_z(\vec {\xi }) = {\hat{b}}_{\Theta _2}^-(\vec {\xi }) \\ &{}\sin \theta _3 \, \cos \phi _3 \, {\hat{m}}_x(\vec {\xi }) + \sin \theta _3 \, \sin \phi _3 \, {\hat{m}}_y(\vec {\xi }) + \cos \theta _3 \, {\hat{m}}_z(\vec {\xi }) = {\hat{b}}_{\Theta _3}^-(\vec {\xi }) \end{array}\right. } \end{aligned}$$

One can check that the corresponding normal vectors $($-$1/\sqrt{2}, \, 0, \, 1/\sqrt{2})$, (0.4695, -0.8133, 0.3437), and (-0.2953, -0.5116, 0.8069) are linearly dependent with rank two. Consequently, Eq. 12 does not have a unique solution. It verifies that in-plane rotations are not sufficient for the reconstruction of the magnetization ${\textbf {M}}$.

This analysis differs from Norton^50^ and Phatak’s theoretical development^25^, which analyzes the reconstruction of the magnetic vector field instead. In that case, the authors can find a linearly independent system of three equations to separate the frequency signals of the magnetic vector field ${\textbf {B}}$. While the first two constraints are obtained from rotations, the last constraint is found by Gauss’s law $\nabla \cdot {\textbf {B}}= 0$ (since ${\textbf {B}}$ is divergence-free). The magnetization vector field is not divergence-free but has another important property: the magnetization field can only exist in a magnetic material. Hence, one can utilize a support (defined as a 3D boundary of the magnetic material) as the necessary and complimentary constraint for the completeness of a magnetization reconstruction algorithm.

Furthermore, for the case of micro-magnetic and no external dynamics at the boundary, we can add in the boundary condition that the gradient of the magnetization is parallel to surface^55–57^, i.e. $\frac{\partial {\textbf {M}}}{\partial {\textbf {n}}} = 0$. In practice, since the support and boundaries are difficult to get computed exactly, one should not enforce the constraint rigidly but relax it as a regularizer instead. We add this regularizer to the minimization (7):

13 $$\begin{aligned} \min _{{\textbf {M}}} \, \varepsilon ({\textbf {M}})&= \frac{1}{2}\sum _{\Theta } \big \Vert \alpha _{\Theta } \, \Pi _{\Theta } (M_x) + \beta _{\Theta } \, \Pi _{\Theta } (M_y) + \gamma _{\Theta } \, \Pi _{\Theta } (M_z) - b_{\Theta }^- \big \Vert ^2 + \frac{\epsilon }{2} \Vert \nabla {\textbf {M}}\cdot {\textbf {n}}_{\partial \Omega } \Vert ^2_{\partial \Omega } \end{aligned}$$

For the regularizer part, ${\textbf {n}}_{\partial \Omega } = (n_1, n_2, n_3)$ is the normal vector to the boundary surface $\partial \Omega$ of the magnetic sample and $\epsilon$ is the regularizer parameter. The regularizer term $\Vert \nabla {\textbf {M}}\cdot {\textbf {n}}\Vert ^2_{\partial \Omega }$ only takes places on the boundary and should not affect the magnetization within the magnetic structure. In further expansion, we can write the regularizer explicitly as $\Vert \nabla {\textbf {M}}\cdot {\textbf {n}}_{\partial \Omega } \Vert ^2_{\partial \Omega } = \big \Vert n_1\frac{\partial M_x}{\partial x} + n_2 \frac{\partial M_y}{\partial y} + n_3 \frac{\partial M_z}{\partial z} \big \Vert ^2_{\partial \Omega }$. $\epsilon$ is tunable and should be small for non-exact support. We can even ignore this regularizer (or set $\epsilon =0$) when the support cannot be computed accurately. In contrast, we can choose large $\epsilon$ for larger effect if the exact support is given. The final gradient will get computed with the extra term as below:

14 $$\begin{aligned} \frac{\partial \varepsilon }{\partial M_x} = \sum _{\Theta } \alpha _{\Theta } \, \Pi _{\Theta }^T \Big ( \Pi _{\Theta } \big ( \alpha _{\Theta } M_x + \beta _{\Theta } \, M_y + \gamma _{\Theta } \, M_z \big ) - b_{\Theta }^- \Big ) + \epsilon \, n_1 \, \frac{\partial ^T}{\partial x} \Big ( n_1\frac{\partial M_x}{\partial x} + n_2 \frac{\partial M_y}{\partial y} + n_3 \frac{\partial M_z}{\partial z} \Big )_{\partial \Omega } \end{aligned}$$

Next, we discuss the robustness of each magnetization component reconstruction $M_x$, $M_y$, and $M_z$ concerning the in-plane rotation angles $\phi$. The linear constraint for in-plane rotations as in Eq. 12 reveals that the x and y components are coupled by linear factors $\sin \theta \, \cos \phi$ and $\sin \theta \, \sin \phi$ while the linear factor of z is only $\cos \theta$. So, the x and y parts are coupled at a higher degree than the z component. As a result, the z component will get decoupling easier and yield a high-quality reconstruction than the other two. Assuming that two in-plane rotation $\phi _1$ and $\phi _2$ are chosen, then these two angles should be chosen equally distanced on half of the unit circle to improve the robustness of the reconstruction. We can choose $\phi _1=0^\circ$ and $\phi _2=90^\circ$ as a simple option.

Side rotations can improve the robustness of the x and y components, but that approach is experimentally infeasible. The summary of our analysis is shown below:

1. In-plane rotations are necessary but not sufficient to decouple the Fourier coefficients of the three magnetization components.
2. Other constraints, such as support and boundary constraints, and regularizers, need invoking, if possible, for highly accurate reconstruction.
3. The z component gets reconstructed with higher quality than the x and y components in in-plane rotation systems.

With the help of a support constraint, we will show that highly accurate vector tomography reconstruction can be obtained numerically with in-plane rotations. Finally, to end this session, we present the experimental scheme of our X-ray vector tomography in Fig. 1. In this case, diffraction patterns are collected, and the projections are obtained via ptychography algorithms^58–60^ before RESIRE-V is used to reconstruct the magnetization.

## Vector tomography reconstruction of simulated data

In this simulation, the sample is a meta-lattice with size of $100 \times 100 \times 100$ pixels. The signals of the magnetization make up around $1.65\%$ of the total signal. Two tilt series from two in-plane rotations where $\phi =0^\circ$ and $90^\circ$ are inspected. For each tilt series, 45 projections of each left and right polarization $P_{\Theta }^+$ and $P_{\Theta }^-$ are generated in the range of $[-66^\circ ,66^\circ ]$ with an increment of 3 degrees. So totally, we generate 180 projections with size $100 \times 100$ pixels. To make it realistic, we add Poisson noise to the projections by selecting a flux of 4*e*8 photons. This flux yields an SNR of 200 and less than $1\%$ noise. Reconstructing the non-magnetic part is not the interest of this research. However, we need to assume that the support of the non-magnetic part is given since it plays an essential role in the reconstruction of the magnetization ${\textbf {M}}$.

Next, we take the left and right projection difference $b_{\Theta }^- = \frac{1}{2}( P_{\Theta }^+ - P_{\Theta }^-)$. Since the magnetic part only makes up a fraction of the total signal, its SNR is much smaller than that of the non-magnetic part. The SNR of the projection difference is approximately $1.65\% \times 200 = 3.3$, which is quite small. The high noise level in the projection differences causes the reconstruction of the magnetization ${\textbf {M}}$ to be less robust than the scalar one. Assuming the noise level in the non-magnetic part stays the same, the robustness of the reconstruction will decline as the magnetization signals decrease relative to the non-magnetic signals.

**Figure 3:**
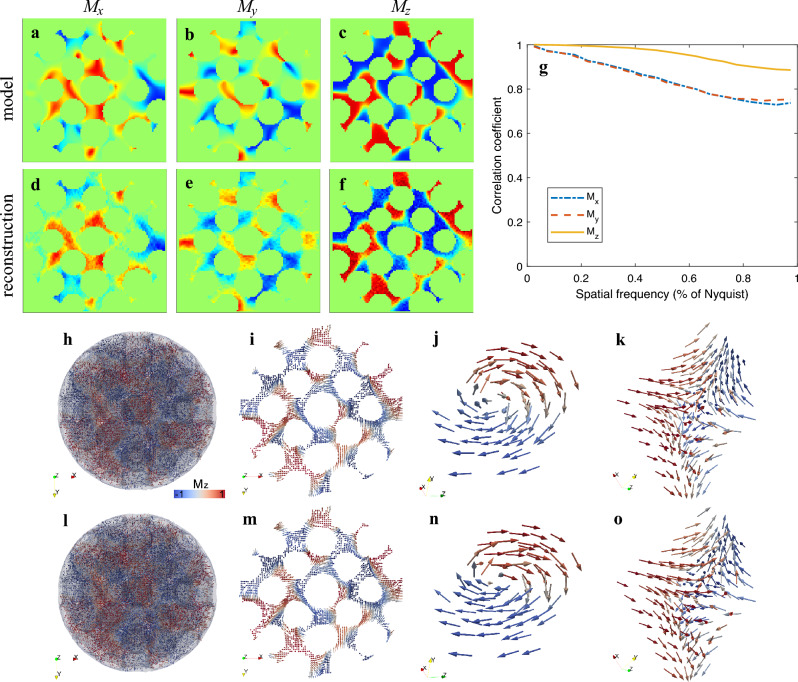
Vector tomography reconstruction of simulated data. (**a-f**) Three magnetic components at the central slice in the z direction of the model (**a–c**) and vector tomography reconstruction (**d–f**) from the simulated data where the normalized cross correlations are 94.1%, 93.8% and 99.1% for $M_x$, $M_y$ and $M_z$ respectively. (**g**) Fourier shell correlation of the three magnetic components, also confirming that the z component has higher quality than the x and y components. (**h–k**) 3D magnetization vector field of the model, including the overall vector field (**h**), the central slice along the z direction (**i**), two topological defects with positive charge (**j**) and negative charge (**k**), where the colors represent the different directions of the vectors. (**l–o**) Reconstructed 3D magnetization vector filed, including the overall vector field (**l**), the central slice along the z direction (**m**), two topological defects with positive charge (**n**) and negative charge (**o**), which are in good agreement with (**h–k**).

Now we use our algorithm to reconstruct the three magnetization components $M_x$, $M_y$, and $M_z$. The model and result are shown in Fig. 3. Since the support constraint is enforced, that is, the magnetization field only appears in the magnetic material. In addition, since we use two tilt series at $\phi =0^\circ$ and $\phi =90^\circ$, the missing wedge artifact does not significantly affect the reconstruction. Fig. 3d–f, shows $M_x$, $M_y$, and $M_z$ components in the central slice along the z-axis, which are in good agreement with the model. The qualities of reconstructions in all directions are comparable and as good as the model (Fig. 3a–c). To quantify the vector tomography reconstruction, we calculate the Fourier shell correlation of the three components between the model and the reconstruction (Fig. 3g). The large correlation coefficients indicate the excellent quality of the vector tomography reconstruction. Additionally, we observe that the reconstructed $M_z$ has higher quality than $M_x$ and $M_y$, which is consistent with our analysis. Figure 3h–i shows the 3D magnetization vector field of the reconstruction and the central slice along the z-axis, respectively, which agree with the model (Fig. 3l,m). We also plot two topological defects with one positive topological charge (Fig. 3j) and the other negative charge (Fig. 3k), both of which are in accordance with the model (Fig. 3n,o). All these analyses confirm that RESIRE-V can reconstruct a high-quality 3D vector field from multiple tilt series each with a limited number of projections with a missing wedge.

**Figure 4:**
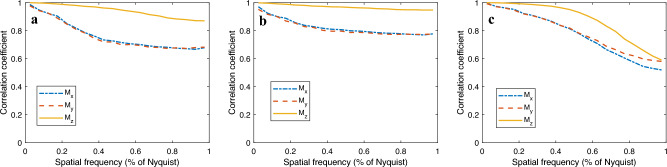
Fourier shell correlation (FSC) of the vector tomography reconstruction of the simulated data using regularziers: (**a**) $l_2$ norm $\Vert {\textbf {M}}\Vert$, (**b**) squared $l_2$ norm $\Vert {\textbf {M}}\Vert ^2$ and (**c**) squared $l_2$ norm of the gradient $\Vert \nabla M_x\Vert ^2 + \Vert \nabla M_y\Vert ^2 + \Vert \nabla M_z\Vert ^2$. The high-frequency information improves under the squared $l_2$ norm regularizer while the low-frequency information decreases. In contrast, the squared $l_2$ norm of the gradient enhance the low-frequency information via smoothing but destroy some important high-frequency information. The $l_2$ norm regularizer worsens the signal since it is not a proper regularizer for this case.

To verify the effectiveness of the regularizers, we add three more reconstructions with regularizers: the first with $l_2$ norm $\Vert {\textbf {M}}\Vert$, the second using $l_2$ norm squared $\Vert {\textbf {M}}\Vert ^2$ and the third with $l_2$ norm squared of the gradient $\Vert \nabla M_x \Vert ^2 + \Vert \nabla M_y \Vert ^2 + \Vert \nabla M_z \Vert ^2$. The reconstructions are shown in Supplementary Fig. 2 and the FSC curves are shown in Fig. 4. The $l_2$ norm regularizer (Fig. 4a) does not help due to the high density of the vector field in this case. The $l_2$ norm squared regularizer (Fig. 4b) helps to improve the high-frequency information, but slightly reduces the low frequency information. In contrast, the squared of the $l_2$ norm of the gradient regularizer (Fig. 4c) improves the low-frequency correlation but decreases the high-frequency correlation.

Other regularizers can be considered such as total variation (TV)^13^ and Markov random field-based regularizer, which have been applied in model-based iterative reconstruction^14^. One should proceed with regularizers with caution. Regularizers can only be applied when prior knowledge is given.

## Vector tomography reconstruction of an experimental data of a ferromagnetic meta-lattice

**Figure 5:**
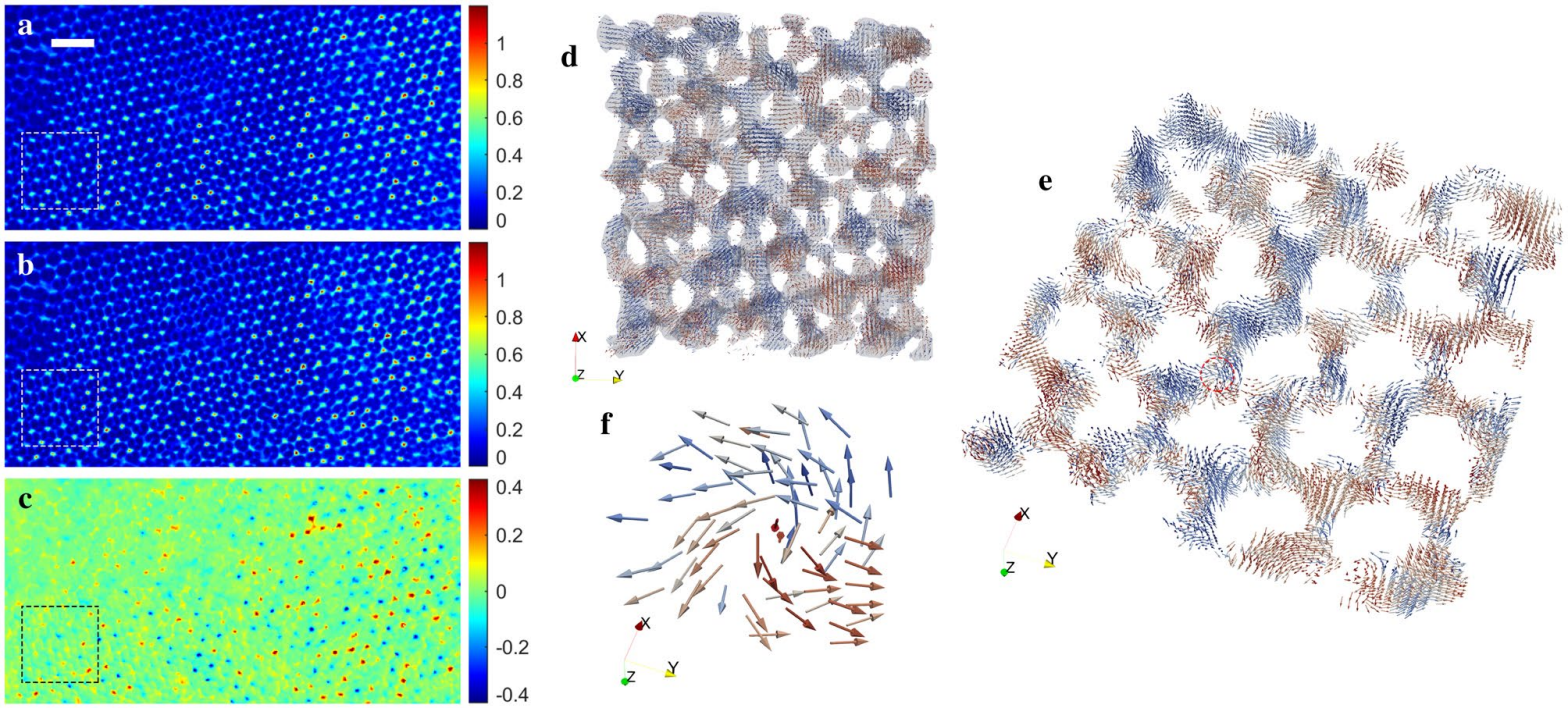
3D reconstruction of the magnetization vector filed in a ferromagnetic meta-lattice. (**a, b**) Representative project with left (**a**) and right (**b**) polarization at $0^\circ$. (**c**) The magnetic contrast projection at the $0^\circ$, which is the difference of the left and right polarization projections. (**d**) 3D magnetization vector field in the square with dashed lines in (**a–c**), where the colors represent the different directions of the vectors. (**e**) A thin layer of (**d**). (**f**) A representative topological defect with positive charge. Scale bar 200 nm.

The ferromagnetic meta-lattice consists of 60 nm silica nanoparticles forming a face-centred cubic structure infiltered with nickel. The vector tomography experiment was conducted at the COSMIC beamline at the Advanced Light Source, Lawrence Berkeley National Lab. Circularly polarized x-rays of left- and right-helicity were used to achieve differential magnetic contrast based on x-ray magnetic circular dichrosim^33,61^. The x-ray energy was set as 856 eV, slightly above the nickel $L_3$ edge. Three in-plane rotation angles ($0^{\circ }$, $120^{\circ }$ and $240^{\circ }$) were chosen, and tilt ranges is from $-62^{\circ }$ to $+61^{\circ }$ for each in-plane rotation angle. At each tilt angle, 2D diffraction patterns were reconstructed using the regularized ptychographic iterative engine, producing two projections with left and right polarization at each tilt angle (Fig. 5a,b). The scalar tomography reconstruction was performed from three sets of tilt series by summing each pair of the oppositely polarized projections, from which a 3D support was obtained to separate the magnetic materials from the non-magnetic region. For the vector tomography reconstruction, the difference of the left- and right-circularly polarized projections was calculated (Fig. 5c), producing magnetic contrast projections of three independent tilt series. Using RESIRE-V with the support, we reconstructed the 3D magnetization vector field. Figure 5d shows the 3D vector field of the magnified square region with dotted lines in Fig. 5a–c, where the colors represent the different directions of the vectors. A thinner slice of the magnified region and a topological defect with a positive charge are shown in Fig. 5e,f, respectively. A more detailed analysis of the 3D magnetization vector field and the topological defects in the ferromagnetic meta-lattice can be found elsewhere^41^.

## Conclusion

We present the mathematical formulation and implementation of RESIRE-V, an iterative algorithm for the 3D reconstruction of the vector field. RESIRE-V requires the acquisition of multiple tilt series of projections and the algorithm iterates between these projections and a 3D structure by using a forward and a backward step. The forward and backward steps consist of the Radon transform and a linear transformation, respectively. Our analysis indicates that incorporating a 3D support to separate the magnetic region from a non-magnetic region can help RESIRE-V achieve accurate and robust reconstruction of the 3D vector field. To validate RESIRE-V, we perform a numerical simulation of the 3D magnetization vector field in a meta-lattice. Using only two tilt series and a support, we reconstruct the 3D vector field with high accuracy. We also observe that the reconstructed z component has higher quality than the x and y components, which is consistent with our mathematical analysis. Finally, we apply RESIRE-V to an experimental data set of a ferromagnetic meta-lattice, consisting of three tilt series with different in-plane rotation angles. Each tilt series has two sets of projections with left and right polarization. By using a support constraint, we reconstruct the 3D magnetization vector field inside the ferromagnetic meta-lattice, showing topological defects with positive and negative charges. We expect that RESIRE-V can be a general vector tomography method for the 3D reconstruction of a wide range of vector fields.

### Supplementary Information

Supplementary Information.

## Data Availability

The MATLAB source codes of RESIRE-V and the simulated and experiment data of the meta-lattice are available at the github repository https://github.com/minhpham0309/RESIRE-V.
