# Supplementary material for: Real space iterative reconstruction for vector tomography (RESIRE-V)
Source: Sci Rep. 2024 Apr 25;14:9541. doi: 10.1038/s41598-024-59140-1 (PMC11045750; doi:10.1038/s41598-024-59140-1)
Supplement: Supplementary file 1 — Supplementary Information. [file 41598_2024_59140_MOESM1_ESM.pdf]

Real space iterative reconstruction for vector tomography (RESIRE-V)

Minh Pham[1,2,3,*], Xingyuan Lu[1,4], Arjun Rana[1], Stanley Osher[2,3], and Jianwei Miao[1,*]

[1]Department of Physics & Astronomy and California NanoSystems Institute, University of California, Los Angeles, CA 90095, USA
[2]Department of Mathematics, University of California, Los Angeles, CA 90095, USA
[3]Institute of Pure and Applied Mathematics, University of California, Los Angeles, CA 90095, USA
[4]School of Physical Science and Technology, Soochow University, Suzhou 215006, China
[*]minhrose@ucla.edu; miao@physics.ucla.edu

Supplementary Information

Step-size analysis

After the gradient is established, we need to perform a step-size analysis, showing and proving a step-size that works. The iterations will then be guaranteed to converge to a solution as desired. To proceed with our goal, we approximate the Lipchitz constant L of the gradient $\nabla\varepsilon$, i.e. find L such that the following inequality holds:

$$\left\|\nabla\varepsilon(\mathbf{M}_1)-\nabla\varepsilon(\mathbf{M}_2)\right\| \leq L\left\|\mathbf{M}_1-\mathbf{M}_2\right\| \quad \forall\, \mathbf{M}_1,\mathbf{M}_2 \tag{1}$$

The step-size then can be chosen to be $1/L$ to guarantee a convergence. First, let decompose the error metric function $\varepsilon(\mathbf{M})$ into a sum of N sub-error metric functions, which correspond to N projections, i.e. $\varepsilon(\mathbf{M})=\sum_\theta \varepsilon_\theta(\mathbf{M})$ where

$$\varepsilon_\theta(\mathbf{M})=\frac{1}{2}\left\|\alpha_\theta\,\Pi_\theta(M_x)+\beta_\theta\,\Pi_\theta(M_y)+\gamma_\theta\,\Pi_\theta(M_z)-b_\theta^-\right\|^2 \tag{2}$$

It suffices to show the Lipchitz constant L_θ of the sub-error metric function $\varepsilon_\theta(\mathbf{M})$. The Lipchitz constant L of the sum function $\varepsilon(\mathbf{M})$ can be derived via some algebra techniques and triangle inequalities after L_θ is obtained.

Let $N_x\times N_y\times N_z$ be the size of each reconstructed magnetic component M_x, M_y and M_z. We further assume that M_x, M_y, M_z and $\mathbf{M}$ are vectorized into 1D vectors and that $\mathbf{M}$ stacks all M_x, M_y and M_z together in this respective order. The purpose of this assumption is for matrix analysis only. Hence we can decompose $\left\|\nabla\varepsilon_\theta(\mathbf{M})\right\|^2$ into a sum as follow:

$$\left\|\nabla\varepsilon_\theta(\mathbf{M})\right\|^2=\left\|\frac{\partial\varepsilon_\theta}{\partial M_y}(\mathbf{M})\right\|^2+\left\|\frac{\partial\varepsilon_\theta}{\partial M_z}(\mathbf{M})\right\|^2+\left\|\frac{\partial\varepsilon_\theta}{\partial M_z}(\mathbf{M})\right\|^2 \tag{3}$$

To show an upper bound of $\left\|\frac{\partial\varepsilon_\theta}{\partial M_x}(\mathbf{M})\right\|^2$, we use a sequences of triangle inequalities (or Cauchy–Schwarz):

$$\begin{aligned}
\left\|\frac{\partial\varepsilon_\theta}{\partial M_x}(\mathbf{M})\right\|^2 &\leq \left\|\alpha_\theta\left(\alpha_\theta\Pi_\theta^T\Pi_\theta(M_x)+\beta_\theta\Pi_\theta^T\Pi_\theta(M_y)+\gamma_\theta\Pi_\theta^T\Pi_\theta(M_z)\right)\right\|^2\\
&\leq 3\,\alpha_\theta^2\left\|\Pi_\theta^T\Pi_\theta\right\|^2\left(\alpha_\theta^2\left\|M_x\right\|^2+\beta_\theta^2\left\|M_y\right\|^2+\gamma_\theta^2\left\|M_z\right\|^2\right)\\
&\leq 3\,\alpha_\theta^2\,N_z^2\max\{\alpha_\theta^2,\beta_\theta^2,\gamma_\theta^2\}\left\|\mathbf{M}\right\|^2
\end{aligned} \tag{4}$$

Here, we use the result $\left\|\Pi_\theta^T\Pi_\theta\right\|\leq N_z$ from elsewhere[2]. The Lipchitz constant L_θ will be obtained by just summing all three inequality, and taking the square root:

$$\left\|\nabla\varepsilon_\theta(\mathbf{M}_1)-\nabla\varepsilon_\theta(\mathbf{M}_2)\right\|\leq\sqrt{3}N_z\sqrt{\alpha_\theta^2+\beta_\theta^2+\gamma_\theta^2}\,\max\{|\alpha_\theta|,|\beta_\theta|,|\gamma_\theta|\}\left\|\mathbf{M}_1-\mathbf{M}_2\right\| \tag{5}$$

Using the fact that $\alpha_\theta^2+\beta_\theta^2+\gamma_\theta^2=1$, we can further simplify $L_\theta=\sqrt{3}N_z\max\{|\alpha_\theta|,|\beta_\theta|,|\gamma_\theta|\}$. Note that $\gamma_\theta=0$ for $\theta=0$. We finally obtain an approximation of the Lipchitz constant $L=\sqrt{3}\,n\,N_z$ and the proof is done. Once can choose the step size $t=1/L$ to maximize the convergence speed. In practice, t could be slightly larger than $1/L$ and the algorithm still converges.

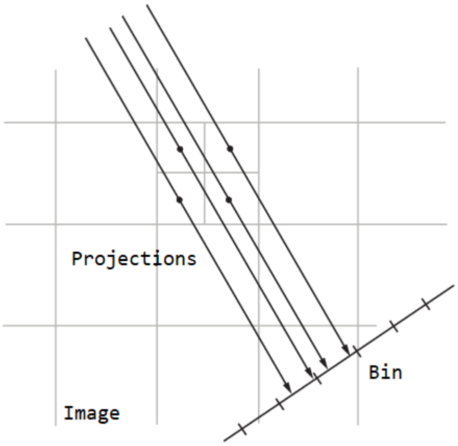

Supplementary Figure 1. Illustration of the Radon transform in the 2D case from Matlab[1]: The algorithm first divides image pixels into four sub-pixels and projects them onto a 2D plane separately. The value of each sub-pixel is distributed proportionally to two nearest neighbors, according to the distance between the projected location and the pixel centers. The transpose of the Radon transform follows the same idealogy in the reverse order. According to the distance between the projected location and the pixel centers, the two nearest neighbors to a projection sub-pixel proportionally contribute their values to the sub-pixel.

References

1. Mathworks.com. Calculate radon transform and display plot. http://www.mathworks.com/help/images/ref/radon.html. Accessed: 2022-02-06.

2. Pham, M., Yuan, Y., Rana, A., Osher, S. & Miao, J. Accurate real space iterative reconstruction (RESIRE) algorithm for tomography. *Sci. Reports* **13**, 5624 (2023).

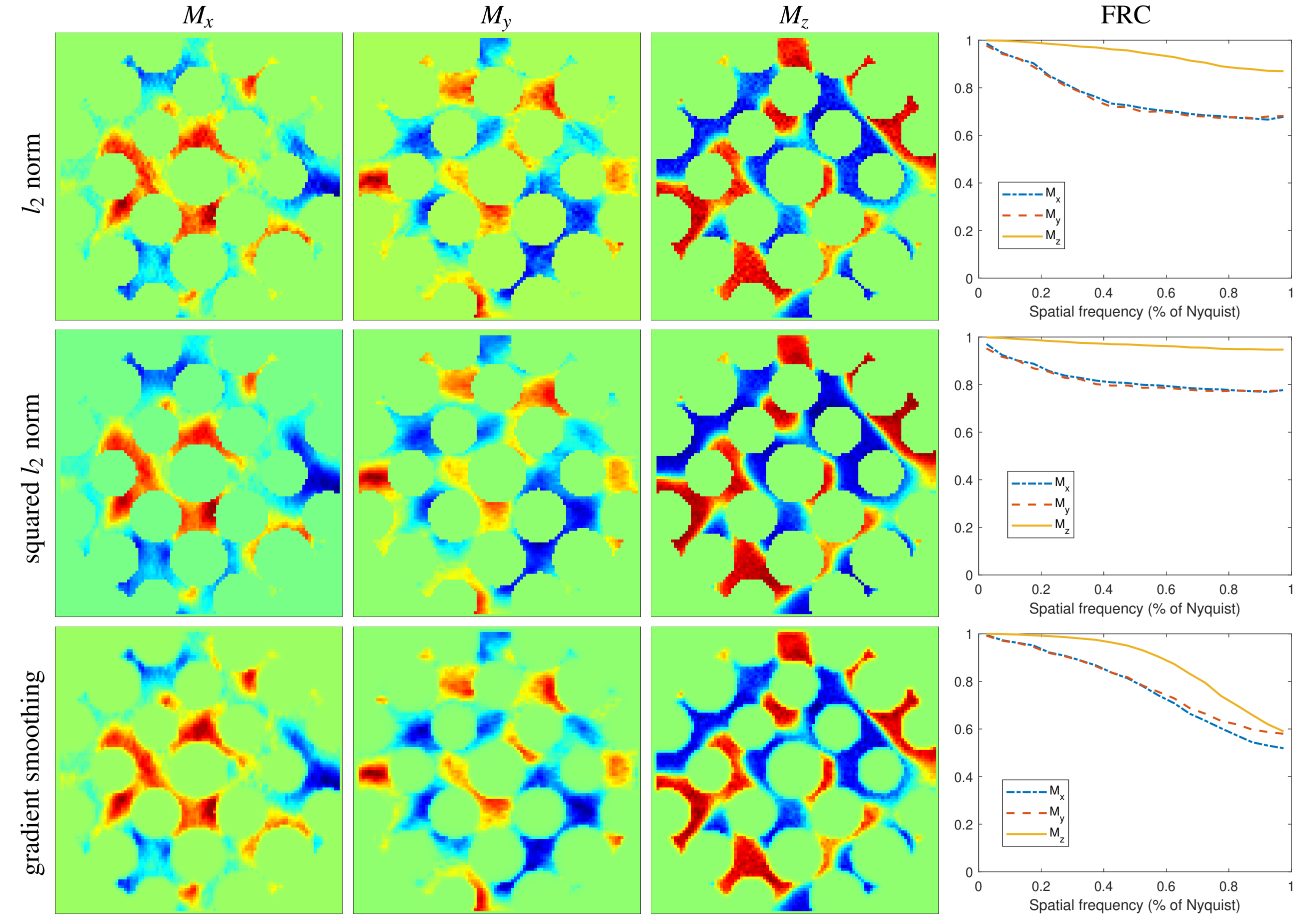

Supplementary Figure 2. Vector tomography reconstruction of simulated data using regularizers such as l_2 norm $\|\mathbf{M}\|$ (first row), squared l_2 norm $\|M\|^2$ (second row), and squared l_2 norm of the gradient (gradient smoothing) $\|M_x\|^2 + \|M_y\|^2 + \|M_z\|^2$ (third row). On each row are the central slices of the three magnetization components M_x, M_y, and M_z and the FRC curve. The squared l_2 norm regularizer improves high-frequency information while slightly reducing some low-frequency information. The reconstruction using gradient smoothing regularizer exhibits smooth features. As a result, the low-frequency information is enhanced while the high-frequency information decreases dramatically. The l_2 norm regularizer results in sparse signal which is not desired in this case.
